# Design and Evaluation of Personalized Motivational Messages by a Virtual Agent that Assists in Post-Traumatic Stress Disorder Therapy

**DOI:** 10.2196/jmir.9240

**Published:** 2019-03-27

**Authors:** Myrthe L Tielman, Mark A Neerincx, Willem-Paul Brinkman

**Affiliations:** 1 Delft University of Technology Delft Netherlands; 2 Netherlands Organisation for Applied Scientific Research (TNO) Soesterberg Netherlands

**Keywords:** mental health, motivation, trust, user-computer interface, PTSD, computer assisted therapy

## Abstract

**Background:**

Systems incorporating virtual agents can play a major role in electronic-mental (e-mental) health care, as barriers to care still prevent some patients from receiving the help they need. To properly assist the users of these systems, a virtual agent needs to promote motivation. This can be done by offering motivational messages.

**Objective:**

The objective of this study was two-fold. The first was to build a motivational message system for a virtual agent assisting in post-traumatic stress disorder (PTSD) therapy based on domain knowledge from experts. The second was to test the hypotheses that (1) computer-generated motivating messages influence users’ *motivation to continue* with therapy, *trust in a good therapy outcome*, and the *feeling of being heard* by the agent and (2) personalized messages outperform generic messages on these factors.

**Methods:**

A system capable of generating motivational messages was built by analyzing expert (N=13) knowledge on what types of motivational statements to use in what situation. To test the 2 hypotheses, a Web-based study was performed (N=207). Participants were asked to imagine they were in a certain situation, specified by the progression of their symptoms and initial trust in a good therapy outcome. After this, they received a message from a virtual agent containing either personalized motivation as generated by the system, general motivation, or no motivational content. They were asked how this message changed their *motivation to continue* and *trust in a good outcome* as well as how much they felt they were *being heard by the agent*.

**Results:**

Overall, findings confirmed the first hypothesis, as well as the second hypothesis for the measure *feeling of being heard* by the agent. Personalization of the messages was also shown to be important in those situations where the symptoms were getting worse. In these situations, personalized messages outperformed general messages both in terms of *motivation to continue* and *trust in a good therapy outcome*.

**Conclusions:**

Expert input can successfully be used to develop a personalized motivational message system. Messages generated by such a system seem to improve people’s *motivation* and *trust* in PTSD therapy as well as the user’s *feeling of being heard* by a virtual agent. Given the importance of motivation, trust, and therapeutic alliance for successful therapy, we anticipate that the proposed system can improve adherence in e-mental therapy for PTSD and that it can provide a blueprint for the development of an adaptive system for persuasive messages based on expert input.

## Introduction

Untreated mental disorders account for 13% of the total global burden of disease, and the gap between treatment needed and treatment received is wide [1]. Electronic health (eHealth) can help remove some of the barriers to care for mental health, offering a cost-effective, accessible, and privacy-sensitive solution to mental health problems [2,3]. A broad range of e-mental health systems exists, differing in purpose and domain. Some systems are only meant for monitoring [4], whereas others offer full therapeutic interventions [5,6]. The latter may be classified as behavior change support systems (BCSS), aiming to change unhealthy behavior patterns. Such systems can be developed for a range of different disorders such as substance abuse [7], anxiety [8], depression [9], or post-traumatic stress disorder (PTSD) [10]. For the success of such BCSS for mental health, the users need to stay motivated to use the system, because behavior change is difficult [11]. Motivational messages can, therefore, be a useful addition to e-mental health systems.

In this study, we focus on an eHealth system for PTSD treatment. PTSD patients have experienced one or more traumatic experiences and have symptoms such as intrusive memories, a persistent negative state, and active avoidance of anything related to their trauma [12]. Of the most common types of therapy for PTSD, 1 is cognitive behavioral therapy including an exposure component. During this exposure, patients need to break the pattern of avoidance by actively recollecting their traumatic experiences [13]. The idea behind exposure is that although tension will initially rise, the automatic fear reaction will eventually fade away [14]. This initial tension means, however, that the situation might temporarily get worse, something that is reflected in treatment manuals [13] and patient reports [15]. This potential worsening also highlights the importance of trust and motivation; it is important that patients still believe they will get better if they persevere. Although shown to be effective [16], exposure therapy is therefore also challenging. This means that in an eHealth therapy system for PTSD treatment such as in the study by Tielman et al [10], motivational messages can be particularly useful.

A therapy system for PTSD can present motivational messages in several ways. Increasingly, BCSS for mental health care include virtual agents to present content to patients [17]. These agents are virtual characters that communicate with the users of a system in some way, and they have been applied, for instance, for dementia [18], substance abuse [19], and PTSD [20]. Virtual agents are perceived more positively by users than a text-based interface [21]. Moreover, they have the potential to positively affect treatment compliance and outcome [22]. This implies that virtual agents can successfully influence users simply by being present. Their impact might partially be explained by self-determination theory, which describes the concept of motivation [12]. It distinguishes between extrinsic motivation, coming from external sources, and intrinsic motivation, which stems from an internal drive and is more powerful of the two. Motivation from BCSS is always extrinsic, but extrinsic motivation might be internalized, a process that is supported by relatedness [23]. When a person feels closer in relation to the source of the extrinsic motivation, the motivation is more likely to be internalized. Virtual agents have the potential to increase relatedness more than simple text messages [24], and are, therefore, suitable to present motivational messages.

Besides the presentation mode of motivational messages, their content is also an important factor. An important question when designing this content is how to tailor it to the user so it is sufficiently effective. This tailoring can be done based on several different factors. One possible factor is readiness to change, which is the underlying concept of the transtheoretical model (TTM) [25]. This is a motivational model that identifies 6 stages of change for health behavior, from where people are not ready yet to make a change to where a change has occurred but needs to be maintained. To change behavior, TTM states a person should move from one stage to the next. Motivational interviewing (MI) is a motivational tactic also based on the readiness to change concept, focusing on increasing motivation by highlighting the discrepancies between the current and the ideal situation [26]. In this way, readiness to change is slowly increased. Both MI and TTM have been applied to virtual agents in various applications [27-30]. However, these models are best considered as full motivational strategies as they focus less on the content of individual motivational messages. Moreover, people in different stages of change do not also prefer different motivational messages [31], making this a less suitable factor for tailoring.

Another possible way to tailor motivation to the user is to consider personality traits. Studies have shown that this influences what type of motivational messages is found preferable [32]. Similar results were found for the effect of masculinity and femininity [33]. Interpersonal differences seem to influence what type of motivation is preferred, but effect sizes in these studies are small. Other factors might, therefore, also be useful to tailor messages in an e-mental health system for PTSD. Moreover, studies with personality traits are commonly performed in the general population and are not focused on specific situations. In PTSD therapy, however, motivation is necessary for a specific task. It seems more useful, therefore, to tailor the messages to the specific health situation of the user instead.

To tailor motivational messages for PTSD therapy to the user’s situation, specific parameters to describe a situation need to be established. In the remainder of this paper, *situation* will, therefore, be defined in terms of the *progression of PTSD symptoms* and the user’s *trust in a good therapy outcome.* These 2 parameters can be related to several theories on health behavior and motivation. Protection motivation theory proposes threat appraisal and coping appraisal as main predictors of health behavior [34]. Progression of PTSD symptoms relates to threat appraisal, as progression allows one to predict how successful therapy will be. Trust in a good therapy outcome, on the other hand, relates to coping appraisal, that is, how much a person feels they will be able to handle what is coming. Similar concepts are found in other theories such as the health belief model [35], which describes parameters such as perceived threat, perceived barriers, and self-efficacy.

To actually develop situation-based motivational messages for a virtual agent assisting in PTSD therapy, their specific content needs to be established. Some systems base the content of their motivational messages on concepts from the literature [36]. However, literature appropriate to the domain is not always available. If larger amounts of data are available on the motivational messages used by therapists, machine-learning techniques could, in principle, also be used to classify which messages are used in which situations. However, such data are rare, given the private nature of conversations between therapists and patients. Another way to build a motivational message system is to code human-human interactions for the use of a virtual agent [37]. This assumes, however, that the virtual agent has the same input from the user as a human would. This is usually not the case as it includes visual cues such as facial expression and posture, which are not always available for analysis. A final option to gather content for motivational messages is to rely on experts to write the messages [8]. This results in an appropriate list, but experts need to write many full messages to allow the system to vary its messages. A way to bypass this final problem would be to break up the messages into smaller, categorized statements that can be recombined into a new message. This allows for the generation of more variation in messages and avoids repetitiveness, which is especially desirable for agents used for long-term interactions [38]. Moreover, by also asking experts to write messages for specific situations, the categorized statements in these messages could be matched to those situations in which they are most suitable.

Therefore, the first objective of this paper is to introduce a method for designing a situation-based motivational system in the domain of PTSD therapy based on expert input. However, the question then remains what effect these motivational messages have on users when they are presented by a virtual agent. Therefore, the second objective of this paper is to empirically study the effect of these motivational messages.

The Methods section first presents our vision for the motivational system and introduces clear hypotheses for the empirical study. It then first outlines the methods for actually creating this message system using expert input. Second, it describes the methods of an empirical study, which was conducted to test the effect of personalized motivational messages presented by a virtual agent. The Results section describes the results of this empirical study, and the Discussion section discusses our findings.

## Methods

### Vision

**Figure 1:**
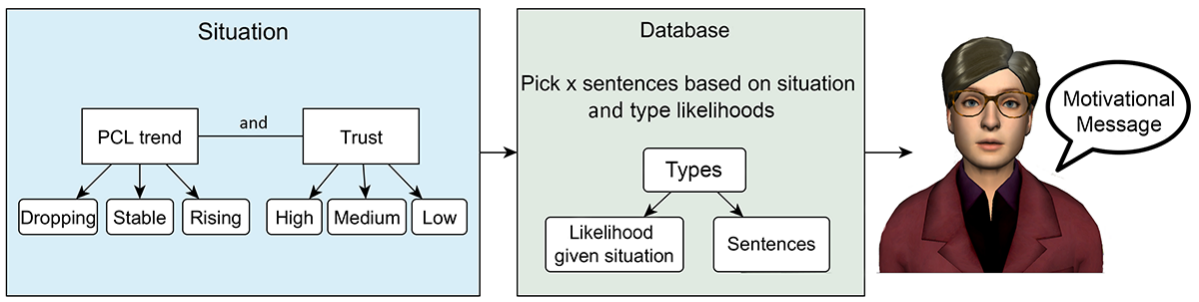
Vision of the situation-based motivational message system. PCL: post-traumatic stress disorder-check-list.

This paper envisions a situation-based motivational message system for a virtual agent for PTSD therapy, as shown in Figure 1. The system generates motivational messages based on a patient’s *situation*, which is defined by the *PTSD symptom trend* and the patient’s *trust in a good therapy outcome* in this context. Such messages consist of different types of statements. These *statement types* are sentences distinguished by their content, for instance, pointing out the situation, asking a question, or giving a compliment. Although questions are not technically statements, we are categorizing them here as such, as we use term “statement” to refer to a sentence that is a part of a motivational message. As the experts knew the virtual agent would never be able to understand any answers to the questions, we interpret these as rhetorical questions. To enable the system to process PTSD symptom trend data, the brief PTSD-check-list (PCL) is used [39]. Through showing PCL scores for past sessions in a graph, *PCL trend* can be defined as dropping (symptoms improve), stable (symptoms stay roughly the same over sessions), or rising (symptoms worsen). *Initial trust in a good therapy* outcome is defined at 3 levels as well and can be either low, medium, or high. This results in a total of 9 possible situations for the system to adapt to.

Given these situations, a suitable *motivational message* can be composed of different *motivational statements*, which are the shorter motivational phrases, categorized by *statement type.* This is done using a database with different types of such motivational statements. The database includes likelihoods that express how suitable a motivational statement of a certain type is, given the situation. By combining specific motivational statements from each type, the system can generate diverse motivational messages suitable for a patient’s situation.

This database, therefore, consists of specific motivational statements, their type, and their suitability for a given situation. This paper envisions that this database can be composed using input from experts familiar with PTSD therapy. Consequently, the first objective is to *build a system that composes motivational messages personalized to user situation based on input from PTSD therapy experts.*

This objective is addressed in the Expert Study section, describing the expert study that was performed and how the eventual motivation system was composed based on the results. In addition, we are also interested in testing the effect of these motivational messages on the people receiving them. The motivational message system is first meant to increase a patient’s *motivation to continue* with therapy. Second, it should also increase their *trust in a good therapy outcome*. Finally, given the importance of therapeutic alliance on the therapeutic process [40], the personalization also serves to increase the patients’ feeling that they are being taken seriously and heard by the virtual agent. The 2 hypotheses were therefore formulated. First, this paper hypothesizes that motivational messages can increase the 3 variables mentioned above. Second, it hypothesizes that by personalizing the motivational messages to a user’s situation, the *motivation*, *trust*, and *feeling of being heard* will improve even further. The following 2 hypotheses were evaluated in a second study performed with users, as presented in the User Study section:

- H_1_: Motivational messages improve trust in a good outcome, motivation to continue, and the feeling of being heard more than messages without motivational content.
- H_2_: Personalized motivational messages as generated by the motivational system improve trust in a good outcome, motivation to continue, and the feeling of being heard more than general motivational messages.

### Expert Study

To achieve the first objective, the motivational message system was created. A database was built including motivational statements, their types, and the likelihood they would be used by experts in a given situation.

#### Participants

A total of 13 therapists (5 male and 8 female) were recruited from 6 different mental health clinics. All experts had professional experience in treating patients with PTSD.

#### Procedure

**Figure 2:**
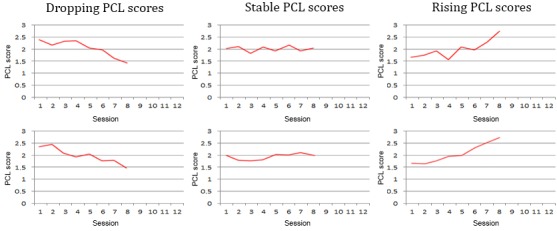
Graphs describing post-traumatic stress disorder symptoms in terms of post-traumatic stress disorder-check-list trend, as presented to the experts in the expert study. PCL: post-traumatic stress disorder-check-list.

All experts were presented with 9 different situations expressed by patient trust in therapy outcome and a graph representing PCL trend. Every situation was presented twice, using 2 different graphs, as shown in Figure 2. This was done to ensure the responses would not reflect 1 specific peculiarity of the graph, but the trend shown therein. For every situation, the expert was asked to write what they would say to the patient to increase their *motivation to continue* and their *trust in a good therapy outcome*. As context, the experts were given the example of the PTSD therapy system presented in the study by Tielman et al. [10]. All situations described a patient in session 8 out of the total 12, which is right in the middle of the most intense phase of treatment. The design of this expert study was approved by the university ethics committee, ID number 134.

#### Expert Answer Analysis

From this study, we received 234 *motivational answers*, that is, motivational messages written by the experts for the situations. These answers were first split into shorter *motivational statements* and categorized to provide them with a *statement type*, after which an analysis was done to calculate the likelihood of the statement types occurring in each situation.

##### Expert Input Categorization

The motivational answers given by the experts were between 1 and 143 words long (Mean 40.40, SD 24.68). The single word Dutch statement “Terecht,” which translates into English as “and you should!,” was used once in a positive situation with high trust. As this word was also included in longer statements, it was categorized as the other “terecht”s, which were part of larger answers and incorporated in the analysis in this manner. Most answers contained several sentences, including a number of messages to the patient. To allow for a more fine-grained categorization, the answers were split into *motivational statements* based on topic. Splitting generally happened after commas or between sentences. This relatively low-inference procedure was as follows: whenever a statement could be categorized in multiple ways and ambiguity could be resolved by splitting, the statement was split. Splitting yielded 844 *motivational statements*. The rounded mean number of *motivational statements* per motivational answer was 3 for the *dropping PCL-high trust*, *dropping PCL-medium trust,* and *stable PCL-high trust* situations, and 4 for the others. All motivational statements were manually sorted into types based on their topic. These *statement types* were not predefined but arose during the analysis. For some, additional subtypes were defined to better describe nuances.

**Figure 3:**
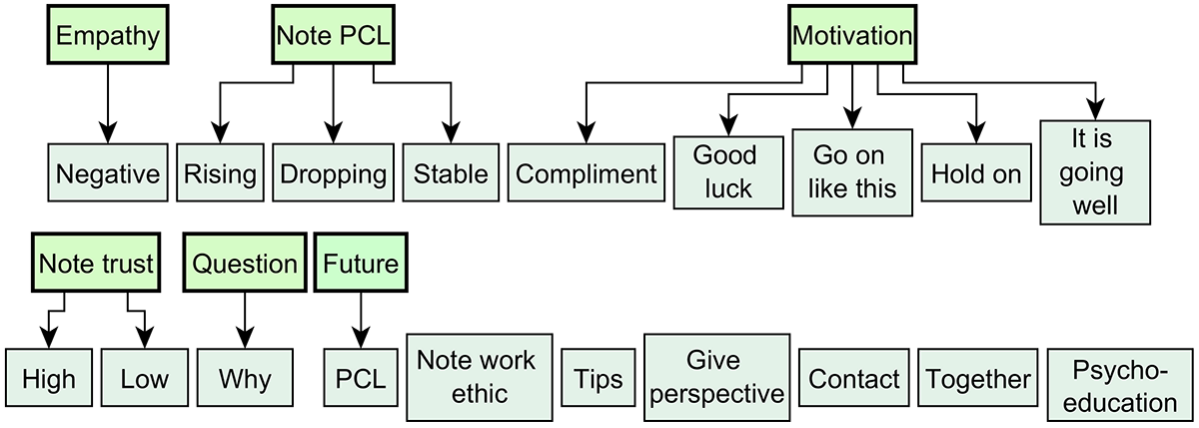
Types of motivational statements found in the expert survey. Thicker bordered green types are super types. PCL: post-traumatic stress disorder-check-list.

After initial categorization, all statement types that included less than 10 motivational statements (in the total set of 844) were removed. After this selection, 97% of the motivational statements were left to be included in the analysis, namely, those with a statement type shown in Figure 3. To analyze the reliability of the coding process, a sample of 32 statements was selected (including 2 full motivational answers for every situation) and rated by a second coder. Comparison between coders showed a substantial interrater reliability (κ=.73, *P*<.001).

It is interesting to note that *medium trust* was not mentioned explicitly by the experts, and thus does not occur in the list of statement types. Although this medium level was included in the situations (defined as “doubts about a good outcome”), in categorizing the answers of experts, no distinction between low and medium trust could be made. Although differences between these situations can be seen in how much other statement types were used, the specific mentions of trust were similar in both situations. This might be because both were interpreted as low trust, one just even lower than the other.

##### Extraction of Likelihood Rates

To calculate the likelihood of a statement type occurring in the motivational answer given a certain situation, the data were analyzed with R 3.3. First, it was noted whether a statement type was present for every motivational answer given by the experts. A multilevel analysis with *expert* as random intercept was done, showing that adding *statement type* as a fixed factor significantly improved the model predicting whether a statement type occurred in the motivational answers (*F*_49__,11638_=88.86; *P*<.001). This indicates that there were differences in how many different statement types were used. Second, an additional analysis showed that the model containing only *statement type* was significantly improved by adding the 3-way interaction effects for *PCL trend*, *trust*, and *statement type* as a fixed factor (χ^2^_3_=1954.80; *P*<.001). This shows that the situation influenced whether a statement type was used in the motivational answer. Therefore, a second multilevel logistic regression was fit for each statement type separately, predicting if a statement type occurred in the motivational answers for that situation. This analysis used *expert* as the random intercept and the 2-way interaction between *PCL trend* and *trust* as the fixed effect. This resulted in the probability of each statement type occurring in a motivational message in a given situation.

These probabilities form the basis of our database. It was supplemented with a list of motivational statements and their type. Motivational statements were included in this list only if the probability for their statement type was above .05 for at least one of the situations. So, those statements with rare statement types were excluded. The list of motivational statements in the system is, therefore, a subset of the motivational statements used in the analysis. All motivational statements were slightly rewritten from how they originally occurred in the motivational answers to ensure they could be used consecutively. Both the full probability table as well as the resulting motivational statements are available online [41].

#### Generating Motivational Messages

Our analysis resulted in a database with motivational statements, their statement types, and their likelihood in any situation. This section describes how this database is used to generate full motivational messages. We have developed an algorithm which selects motivational statements of certain types from the database, given a situation, and which combines them into 1 message. Throughout this section, we will use a running example of generating a message for the situation with *rising PCL* and *low trust.*

First, the length of the motivational message is determined. The motivational answers by the experts revealed that some situations might warrant more motivational statements than others. Therefore, either 3 or 4 motivational statements were combined, depending on the rounded mean number of motivational statements given by the experts in their motivational answers for that situation. For our example message for a situation with *rising PCL* and *low trust,* the motivational message will contain 4 motivational statements.

Second, the statement types are selected. In this, we follow the probability table for the situation. First, any statement types with probabilities above .5 are always included. For our example, this means that the statement types *motivation*, *give perspective,* and *note PCL* will be included.

If any of these statement types with a probability above .5 is a super-type, all subtypes with a probability above .5 are included; if none meet this criterion, one is chosen based on a weighted randomized selection strategy. This strategy entails that all probabilities are added, and every statement type is assigned an interval equal to their probability. By generating a random number between zero and the total sum of the probabilities, the statement type belonging to the interval in which the random number occurred is selected. For our example, the statement types *motivation* and *note PCL* are super types, so a subtype needs to be selected. For *motivation*, none of the subtypes have a probability over .5, so a choice is made with the weighted random selection strategy. For this example, let us say we end up choosing *hold on*. For the *note PCL* type, we do have a subtype with a probability over .5, namely, *rising*, so this will be chosen.

Then, if the expected number of statements is not met after this initial selection, additional statement types are added one by one using the weighted randomized selection strategy. Duplicate statement types are avoided so that if a statement type is already included, it cannot be chosen again. For our example, we still need one more statement type. A final type is chosen with the selection strategy, let us say we end up with the statement type *empathy.*

**Figure 4:**
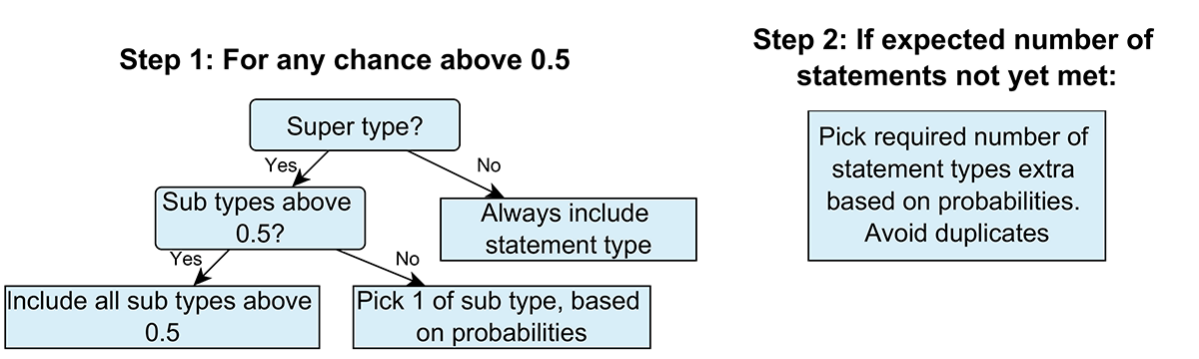
Selection process for choosing the statement types for the motivational message system.

The full selection process for the statement types is described in Figure 4. This process was designed to fit different probability tables. For instance, for our probability table (see the resource by Tielman et al [41]), no statement type has 2 subtypes with probabilities higher than .5, but this algorithm could deal with this situation as well. For application to other probability tables, the only possible change would be the threshold of .5 to always include a statement type. This number was chosen because for our results it meant 2 or 3 statement types are always automatically selected, whereas at least one is always added via the weighted randomized strategy. This ensures variation while also ensuring the most important statement types are always included.

Third, to complete the motivational message, a motivational statement is selected at random for every statement type that is included. This means that even if the statement types for a certain situation are always the same, the specific motivational statements making up the motivational message can differ. The ordering of the motivational statements within the message is inspired by the original motivational answers given by the experts. They are sorted based on statement type, such that *noting the situation* always comes first, followed by *empathy*, *giving perspective*, and statements about tackling things *together*. *General motivation* is always near the end, only followed by statements about the *future*.

For example, this final step means randomly choosing motivational statements from the database for the statement types and putting them in the order: *note PCL-rising, empathy, give perspective,* and *motivation-hold on.* This could result in the following motivational message (translated from the original Dutch):

I see you indicate that your complaints have gotten substantially worse.

note rising PCL

I’m sorry to hear that.

Empathy

However, it’s always hard work before we see any results.

Give perspective

Hold on!

Motivation hold on

### User Study

To test hypotheses 1 and 2, an empirical user study was done. The *personalized motivational messages* as generated by the motivational message system described in the previous section were compared with 2 other *message types*, namely, *general motivational messages* and messages *with no motivational content*.

The motivational message system is designed for PTSD patients following therapy assisted by a virtual agent. However, to evaluate the message system with patients in therapy would mean a full clinical trial, which traditionally mainly focuses on clinical outcome. Instead, eHealth systems are often evaluated in phases [42], and separate components are first tested using proximal outcome measures such as motivation [43,44]. Testing components separately also allows for a clearer picture of exactly what effect a certain manipulation, such as motivational message type (ie, personalized, generic or no motivational message), has on specific outcome variables, such as *motivation to continue*, *trust in a good therapy outcome, or* the feeling the user has of *being heard*. This abstraction strategy is, for instance, also used in game design to reduce surrounding and complicating factors and speed up the process [45]. The evaluation of the motivational message system takes this component-focused approach. As it was tested in isolation instead of incorporated into a full therapy, participants were asked to imagine they were in a certain situation during PTSD therapy, as defined by symptom trend and trust in therapy outcome. One can imagine, however, that people who have experience with PTSD exposure therapy will interpret the situations differently from people who do not. Participants were, therefore, also asked for their experience with PTSD symptoms and exposure therapy so that these factors could be taken into account as potential covariates.

The message system was tested in a 3×3×3 mixed design. Message type (personalized, general, or no motivation) was measured between-subject, whereas the 3 graphs describing PTSD symptom trend and the 3 levels of initial trust in therapy outcome were presented to all participants within-subject. After seeing the situation, participants were presented with a virtual agent presenting a personalized motivational message, a general motivational message, or a message with no motivational content. Following this, participants were asked to indicate changes in their *motivation to continue* and *trust in a good therapy outcome* for that situation. The design of this user study was approved by the university ethics committee as well, ID number 184.

#### Participants

To study the effect of the situation-based motivational message system, participants were recruited via Amazon Mechanical Turk. An a priori power analysis was performed (G*Power 3.1) to determine the necessary number of participants. Given a one-way Analysis of variance (ANOVA) with 3 groups, a medium effect size of 0.25, and power of 0.9, a preferred sample size of 207 was calculated. A total of 529 participants started the experiment. Participants were excluded if they incorrectly answered a control question (to check if they had read all situations and questions; n=189), if they did not complete the survey (n=128), or in case of administrative errors (n=5). Participants were excluded directly after completing the study, which kept running until the required number of 207 included participants was met. Of the excluded participants, 25.5% were in the personalized motivation condition, 27.6% in the general motivation condition, 29.5% in the no motivation condition, and 30.1% stopped before being assigned a condition. Of the 207 included participants, 34.3% were in the personalized motivation condition, 34.3% in the general motivation condition, and 31.4% in the no motivation condition. All participants were aged 18 years and above and native English speakers and received a small monetary compensation for their time. Due to an administrative error, specific age and gender data of participants were not collected.

#### Measures

A total of 5 different measures were collected via questionnaires, 2 of them repeatedly for every situation that was presented. A list of all questions is available online [41].

##### Primary Measures

*Trust in a good therapy outcome* was measured in terms of change. After each situation and message, the question of how much the message of the virtual agent changed *trust in a good therapy outcome* was posed. Answers ranged on an analog scale from −10 (*it decreased a lot*) via the neutral point of 0 (*nothing changed)* to 10 (*it increased a lot)*. This question was repeated at the end of the study, asking for the overall change in *trust*.

*Motivation to continue* was measured similarly to trust, in terms of change caused by the message of the virtual agent, asked for in every situation. The analog scale also ranged from −10 (*it decreased a lot)* via the neutral point of 0 (*nothing changed)* to 10 (*it increased a lot)*, and this question too was repeated at the end asking for the overall change in *motivation*.

*Feeling of being heard* was measured only at the end of the experiment and was included to get a measure of how much the participants felt the agent took them seriously and really heard them. This questionnaire consisted of items 1 and 4 of the short patient satisfaction questionnaire [46], and items 5 and 8 from the trust in physician scale [47]. Questions were slightly adapted to be positively phrased statements. Moreover, 3 additional questions were added to complete the questionnaire, stating that *the virtual agent took you seriously*, the *virtual agent replied appropriately to you*, and the *virtual agent listened to your preferences*.

##### Descriptive Measures

To get an indication of whether participants had experience with PTSD symptoms, a brief version of the PCL was presented at the beginning of the study, including questions 2, 3, 6, 7, 16, and 17. People were asked to keep in mind their most stressful or traumatic experience when answering these questions. Participants were also asked if they had experience in following exposure therapy, and if so for what disorder.

#### Procedure

**Figure 5:**
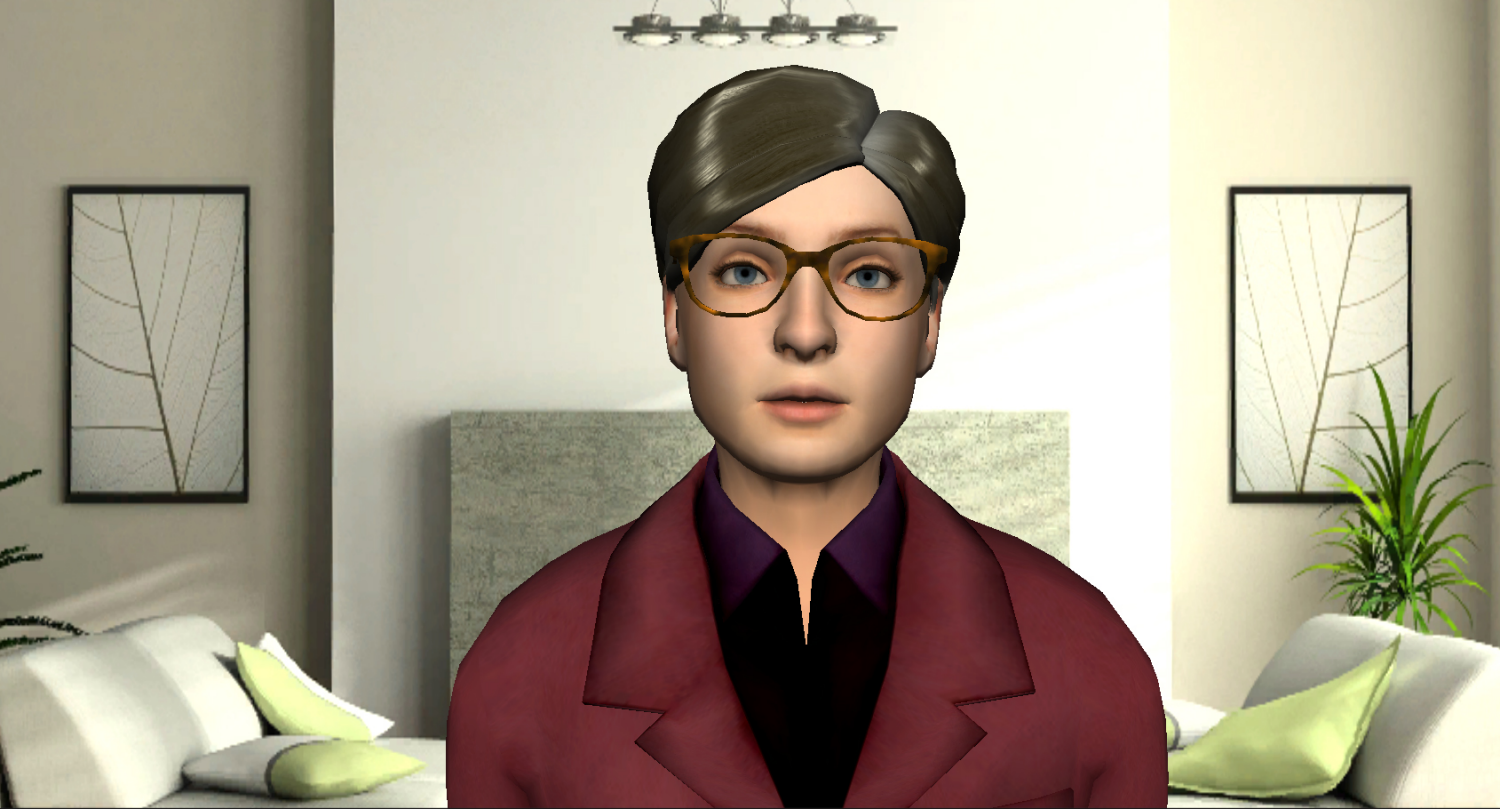
Virtual agent.

After accepting the assignment on Amazon Mechanical Turk, participants were redirected to Qualtrics for the main experiment. They were first presented with an information form outlining the procedure of the experiment and the consent form. Then, they were given the PCL and asked if they had ever followed exposure therapy. Before the main part of the experiment, all participants saw an example situation with a PCL graph that was not reused in the remainder of the experiment, a clip from the virtual agent introducing itself, and the motivation and trust questions. This example included the explicit instruction that a rising PCL trend meant symptoms were getting worse and vice versa. After the example, participants were randomly divided into the 3 conditions (with personalized, general, or no motivation messages). The virtual agent always appeared as a video clip and displayed idle and mouth movement, speaking with a computer-generated text-to-speech voice. The virtual agent is shown in Figure 5.

**Figure 6:**
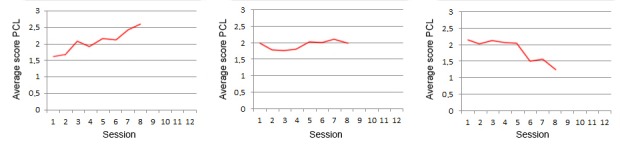
Situations used in the experiment. From right to left: rising post-traumatic stress disorder-check-list, stable post-traumatic stress disorder-check-list, and dropping post-traumatic stress disorder-check-list. PCL: post-traumatic stress disorder-check-list.

In all conditions, participants were asked to repeatedly imagine they were in therapy and had just answered the PCL resulting in the situation shown. All 9 situations representing the combinations of PCL trend (rising, stable, and dropping) and trust level (no trust, doubt, and high trust) were shown in a randomized order. To avoid overfitting, a new set of PCL graphs was used, slightly different than the one shown to the experts. These new graphs used in the user study are shown in Figure 6. After reading the situation, participants could view the message by the virtual agent. In the condition with the personalized motivational messages, 1 of the 3 comments generated by the motivational message system for that particular situation was used, translated from the original Dutch to English. For the condition with general motivational messages, 9 different general motivational messages were written by 3 different experts and translated into English. These general messages were matched with the situations in 3 different ways, such that each general message occurred once for each PCL trend and each trust level in the final set. Participants were randomly presented with 1 of these 3 combinations. The same type of randomization was performed for the messages without motivational content. In this condition, the virtual agent merely thanked the participant for filling in the PCL and announced the continuation of the session, using 9 different formulations. An example of a message from each condition is shown in Table 1. All messages for the different conditions can be found in the resource by Tielman et al [41].

**Figure 7:**
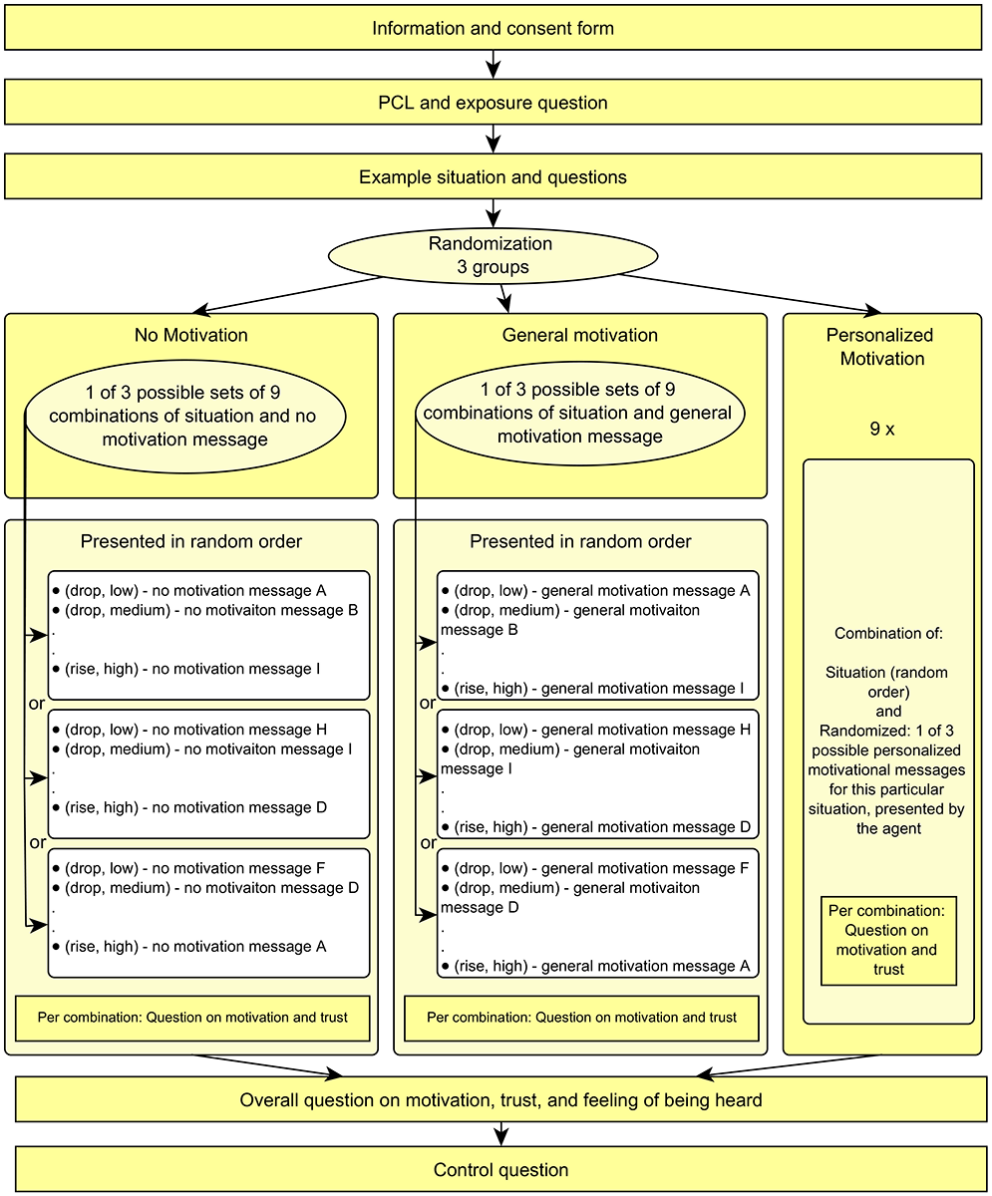
Outline of the user study experiment procedure. PCL: post-traumatic stress disorder-check-list.

After the message by the virtual agent, participants were presented with the question of how much their trust in a good outcome and *motivation to continue* had changed. Both the situation and the virtual agent were still visible at this point; so, participants had the option to review both the situation and the message. After all situations, these 2 questions were repeated for the overall experience, and the *feeling of being heard* questionnaire was presented. Figure 7 shows a full outline of the procedure.

#### Data Preparation and Analysis

Data were analyzed with R 3.3; the full data table and analyses are available online [41]. Following the guidelines for the PCL [39], a participant was classified as potentially having PTSD if question 2 or 3 was 3 or higher, question 6 or 7 was 3 or higher, and question 16 or 17 was 3 or higher. Reliability for the *feeling of being heard* scale was high with a Cronbach alpha of .96, so answers to the items of the *feeling of being heard* scale were averaged to form 1 score.

##### Covariates

To examine *potentially having PTSD* and *having experience with exposure* as potential covariate factors in the analysis, ANOVAs were done with these variables as predictors of overall change in *motivation*, *trust*, and *feeling of being heard score*. As these analyses did not find any effects, neither *potential PTSD* nor *exposure experience* were further taken into account as covariates.

**Table 1:** Examples of the different types of messages presented.

Type	Message
Personalized to stable post-traumatic stress disorder-check-list scores and doubts	Your complaints show little consistency. Sometimes they get less, and then they get worse again. That makes sense, you're working on your past and that’s tough. Stick to it now! Let’s see together what you need to continue.
General	You’ve now been working on this treatment for a while. Perhaps you’ve noticed already that your complaints are slowly getting less. It’s important to stick with it now, so we can achieve the largest result.
No motivation	Thank you for filling in the questionnaires. We’ll now continue with the session.

##### Overall Measures

For all overall measures, a linear regression model with only the intercept was fit to establish a deviation from zero. *Type of message* was added to the model to establish if it affected the overall outcome measures. When this analysis showed a significant effect of *type of message*, individual post hoc analyses were run on data containing only 2 message types to test for differences between them.

##### Per Situation

To analyze the data per situation, every situation was treated as a separate data point. A multilevel regression was run with *participant* as random intercept. For both change in *motivation to continue* and *trust in a good outcome*, the null model with only a fixed intercept was compared with model 1, including *message type* (3 levels) as fixed effect. Model 2 built on model 1 and added *PCL trend* as fixed effect; model 3 added *initial trust level*. Further models were built, adding first the 2-way interaction effects and finally the 3-way interaction effect. Post hoc analyses were run on subsets of the data to find the differences between each of the 3 message types. This was done for the 3 possible *PCL trends*, the 3 levels *of initial trust*, and for the 3×3 combination of these measures resulting in all 9 situations.

## Results

### Descriptive Measures

Table 2 shows the descriptive measures including their influence on the overall outcome measures. Scores on PCL show that almost a third of participants showed an indication of having PTSD. A total of 14 participants had experience with exposure therapy. Out of the 7 participants with experience with exposure for PTSD, 5 also showed an indication of PTSD. The other specific disorders mentioned were obsessive-compulsive disorder and both specific and general anxiety disorders. Table 2 also shows that *an indication of PTSD* or *exposure experience* were not found to affect overall change in *motivation*, *trust,* or *feeling of being heard score*. There was, therefore, no indication found that experience with PTSD or exposure therapy influenced the interpretation of the situations and the effect of the messages by the virtual agent. This supports the generalizability of the results to clinical populations.

### Overall Experiences

Table 3 shows the mean values of all overall outcome variables measured at the end of the experiment as well as their deviance from the neutral point of 0. Both change in *motivation* and *trust* were rated significantly above neutral, indicating that overall *motivation* and *trust* improved. The *feeling of being heard* did not deviate significantly from 0. For all 3 outcome measures, linear regression analyses revealed a significant effect for *message type*.

**Figure 8:**
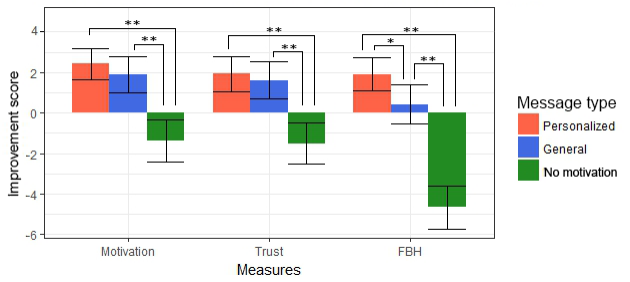
Effect of message type on the overall outcome measures. *P<.05, **P<.01. Error bars indicate 95% Confidence Intervals. FBH: feeling of being heard.

Post hoc analyses revealed the differences between each of the 3 *message types* for the overall variables. Figure 8 visualizes the results from this analysis, showing that *motivational messages* significantly improve overall increase in *motivation* and *trust*. Moreover, the *feeling of being heard* increases significantly not only if a *motivational message* is present but even more if this message is personalized to the participant’s situation. The confidence intervals show that if a *personalized message* was presented, *motivation*, *trust*, and the *feeling of being heard* improved, whereas a message without motivational content always resulted in a decrease.

### Per Situation

Table 4 shows the effects of *message type*, *PCL trend*, and *initial trust* on the outcome measures of *motivation to continue* and *trust in a good outcome*, as measured per situation. This table also includes the effects of the 2- and 3-way interactions.

**Figure 9:**
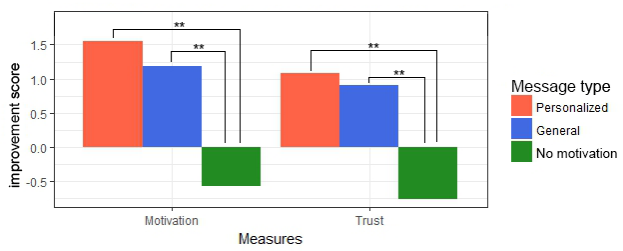
Influence of message type on motivation and trust per situation. **P<.01.

**Figure 10:**
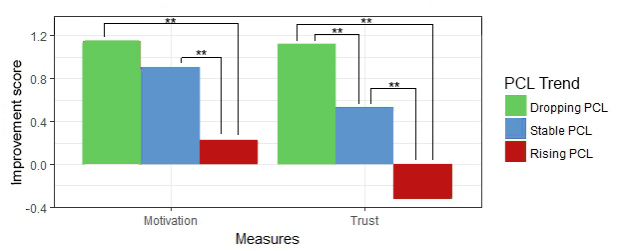
Influence of post-traumatic stress disorder-check-list trend on motivation and trust per situation. PCL: post-traumatic stress disorder-check-list. **P<.01.

**Figure 11:**
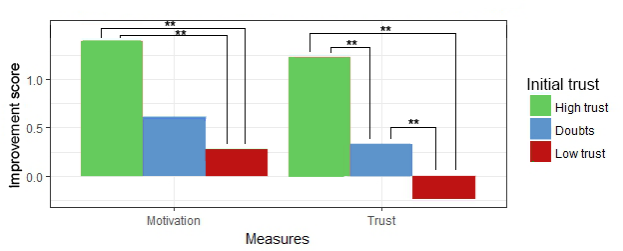
Influence of initial trust on motivation and trust per situation. **P<.01.

This table shows that both *message type* and the 2 situational factors influence *motivation* and *trust* as measured per situation. Figure 9 visualizes the pair-wise comparison of the effect of *message type*, showing that *motivational messages* outperform messages with no motivational content. The differences between the 3 levels of *PCL trend* and *initial trust* are displayed in Figures 10 and 11, respectively, showing how these situational factors affected the outcome measures.

**Figure 12:**
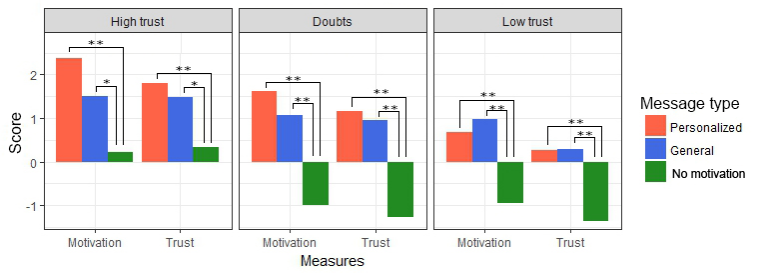
Effect of message type on motivation and trust, per initial trust level. *P<.05, **P<.01.

**Figure 13:**
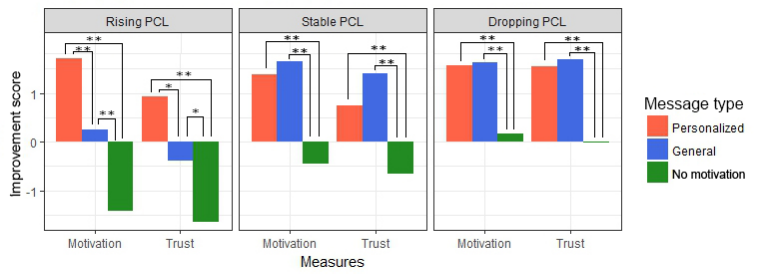
Effect of message type on motivation and trust, per post-traumatic stress disorder-check-list trend. *P<.05, and **P<.01. PCL: post-traumatic stress disorder-check-list.

Besides individual effects, Table 4 also shows the existence of an interaction effect between *message type* and *PCL trend* for both *motivation* and *trust* and a further effect between *message type* and *initial trust* for *motivation*. Given these results, a pair-wise analysis was done comparing each of the message types given the 3 possible values of *initial trust* and *PCL trend*, shown in Figures 12 and 13, respectively. Figure 13 shows that *initial trust* level effects how much *motivation* and *trust* improve. Moreover, if the initial *trust* level is doubtful of low, receiving a message without motivation seems to even reduce *motivation to continue* and *trust in a good outcome*. Figure 13 shows that when the *PCL trend is rising* and the situation, therefore, is getting worse, *personalized motivational messages* significantly *outperformed general motivational messages* in terms of both, *motivation to continue* and *trust in therapy outcome*.

**Figure 14:**
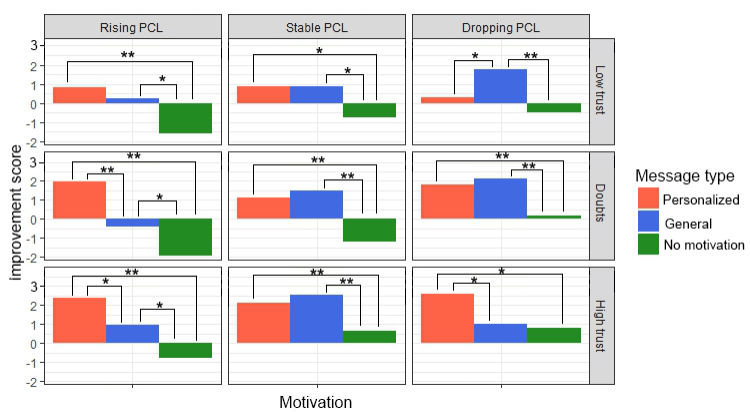
Effect of message type on motivation in all separate situations. *P<.05 and **P<.01. PCL: post-traumatic stress disorder-check-list.

Finally, Table 4 shows the existence of a 3-way interaction effect of *message type*, *initial trust*, and *PCL trend* on *motivation to continue*. Figure 14 shows the effect of *message type* on *motivation to continue* for all 9 situations, combining *PCL trend* and *initial trust level*. These graphs show that the exact effect of *message type* differs per situation. In the situations where symptoms are getting worse, but *initial trust* is doubtful or high, *personalized motivational messages* show an advantage over *general motivational messages*. The same is true for the situation with dropping *PCL trend* and high *initial trust*, which shows not only that personalized motivation is best but also that general motivational messages do not even outperform messages without motivational content. In most other situations, motivational messages of some type outperform messages without motivational content. The only exception is the situation with dropping *PCL trend* and low *initial trust*, where *motivation* is improved most by general motivational messages.

**Table 2:** Descriptive characteristics of participants.

Characteristic	n (%)	Motivation		Trust		Feeling of being heard	
		*F value* (1, 205)	*P* value	*F value* (1, 205)	*P* value	*F value* (1, 205)	*P* value
Post-traumatic stress disorder indication	66 (32)	0.99	.32	0.13	.72	0.04	.83
Exposure experience	14 (7)	1.48	.22	1.18	.28	0.34	.56
Post-traumatic stress disorder	7 (3)	—^a^	—	—	—	—	—
Anxiety	6 (3)	—	—	—	—	—	—
Obsessive-compulsive disorder	1 (0)	—	—	—	—	—	—

^a^Not applicable.

**Table 3:** Description, overall measures, and effects of condition.

Variable	Mean (SD)	Deviance from 0		Effect of message type	
		*t* test (206)	*P* value	*F value* (2, 204)	*P* value
Motivation	1.06 (4.05)	3.76	<.001	20.58	<.001
Trust	0.74 (4.16)	2.56	.011	16.18	<.001
Feeling of being heard	−0.66 (4.83)	−1.96	.051	50.97	<.001

**Table 4:** Influence of situation and feedback type on *motivation to continue* and trust in therapy outcome (n=1863).

Model comparison		*χ*^2^ (degrees of freedom)	*P* value
**Motivation to continue**			
	Add Message type (M_0_ vs M_1_)	29.1 (5)	<.001
	Add PCL^a^ Trend (M_1_ vs M_2_)	27.5 (7)	<.001
	Add initial trust (M_2_ vs M_3_)	40.5 (9)	<.001
	Add initial trust × PCL Trend (M_3_ vs M_4_)	9.4 (13)	.05
	Add message type × PCL Trend (M_4_ vs M_5_)	25.2 (17)	<.001
	Add message type × Initial Trust (M_5_ vs M_6_)	11.2 (21)	.03
	Add message type × Initial Trust × PCL Trend (M_6_ vs M_7_)	17.1 (29)	.03
**Trust in outcome**			
	Add message type (M_0_ vs M_1_)	22.9 (5)	<.001
	Add PCL trend (M_1_ vs M_2_)	55.7 (7)	<.001
	Add initial trust (M_2_ vs M_3_)	59.9 (9)	<.001
	Add initial trust × PCL Trend (M_3_ vs M_4_)	5.3 (13)	.26
	Add message type × PCL Trend (M_4_ vs M_5_)	20.5 (17)	<.001
	Add message type × initial trust (M_5_ vs M_6_)	7.4 (21)	.12
	Add message type × initial trust × PCL trend (M_6_ vs M_7_)	12.7 (29)	.12

^a^PCL: post-traumatic stress disorder-check-list.

## Discussion

### Results of the User Study

The aim of the user study was to test 2 hypotheses about the effect of the (personalized) motivational messages on the *motivation to continue*, *trust in a good therapy outcome*, and the *feeling of being heard by the agent*. The results show that the content of a message influences *motivation to continue*, *trust in a good outcome*, and how much participants *felt heard by the agent*. More specifically, messages with motivational content outperform messages without such content. This confirms hypothesis 1 and is in line with recommendations by experts that motivational messages are relevant [48].

The results regarding hypothesis 2 are a bit more complex. We hypothesized that motivational messages could be improved through personalization with our motivational system. Some findings support this hypothesis. This study shows that personalization can increase how much users feel the agent really *hears them* and takes them seriously. This result is important as it shows that personalization could increase a feeling of relatedness to an agent. Studies on motivation have shown that extrinsic motivation (such as our messages) is less powerful than intrinsic motivation and that for extrinsic motivation to be internalized, a feeling of relatedness is important [23]. More work is needed to identify to what extent the *feeling of being heard* increases relatedness exactly. However, the result that the content of a motivational message can influence this variable is a promising first step to further increase the impact of personalized motivational messages.

Our system aims to improve motivational messages by personalizing them based on the type of situation the user was in. This seems to have worked as these personalized messages were more effective in terms of *motivation to continue* and *trust in a good outcome* in the situation with worsening PTSD symptoms in particular. This is an interesting result as it indicates that personalization is more effective in those situations where motivation is most important.

However, results also indicate that the effect of personalization can only be truly understood if we consider not just the symptoms of the user but also their initial trust levels. These results give a slightly more nuanced picture and do not directly fully support hypothesis 2. For situations with worsening symptoms, personalization improved motivation only when the *initial trust* level was doubtful or high. Therefore, in the situation where symptoms were getting worse and initial trust was low, personalization did not have as much effect. A closer look at the results (see Figure 14) reveals that in this situation, neither type of motivational message (general or personalized) led to a very large improvement in motivation in the first place. An effect between the types of messages was shown, but mostly because a message without motivation led to a decrease in motivation. These results could indicate that if symptoms are worsening and trust is low, a simple message by a virtual agent is not enough to truly improve motivation of the user. Still, these messages might prevent a further decrease in motivation, as was the case with the nonmotivational message. They would, therefore, nevertheless be useful, but should perhaps be supplemented with other types of motivation.

The situations where PTSD symptoms are reducing also show a clear impact of *initial trust level* on the effect of personalization. Although personalization improves motivation in situations with high trust, situations with low trust show an opposite effect, with general motivational messages outperforming personalization. This result is surprising, but 1 possible explanation might be that the personalized messages will point out that scores are dropping, which is already a good sign. If trust level is low, however, people might instead prefer to get reassurance that it is normal to find therapy difficult. Nearly all general motivational messages included this reassurance, whereas the personalized messages for this situation did not, as things were actually going well. This could mean that the messages our experts wrote for this situation were not quite as suitable as believed. Obviously, this result warrants more study into why, exactly, our general messages outperformed those based on the experts’ answers.

Although the overall results, as well as the results from other situations, confirm hypothesis 2, this final result that in 1 situation general motivation outperforms personalized motivation suggests a rejection. A full rejection might, however, be too strong, as it might also be the case that our particular way of personalizing was not effective in this situation. Instead, it might be more fitting to argue that for this specific situation with a reduction of PTSD symptoms and low trust, general motivation is more suitable. To explain why this is the case would require more research.

### Implications for Motivational Models

The evaluation of the personalized motivational messages shows that they are capable of increasing *motivation* and *trust* as well as the *feeling of being heard* by a virtual agent. However, it also shows that personalization is more effective in some situations than in others. These results indicate that it is worthwhile to take a closer look at the motivational model itself and what aspects might be improved in the future.

#### Impact of Situational Variable on Statement Type Choice

The motivational model used 2 input variables to describe user situation, namely, *initial trust* in a good therapy outcome and *PCL trend*. These variables describe aspects of current motivation and symptom progress, respectively. The analysis of our results reveals that to predict which statement types are best, both of these factors are necessary. Moreover, an interaction effect was found, which shows that they cannot be seen separately from each other. However, a closer inspection of the probabilities for each statement type shows that for some, either *PCL trend* or *initial trust* seems more important than the other [41].

Most noticeable is that the *note PCL* statement type has a very high probability for all situations, but whether the subtype is *note stable, note dropping,* or *note rising* is highly dependent on the *PCL trend* and does not seem to be influenced as much by *initial trust*. This makes sense, as this statement type directly refers to the *PCL trend.* For the *note trust* statement type, the story seems a little more complex. Whether trust is noted at all seems dependent on both *initial trust* and *PCL trend*. However, whether *high trust* is noted or *low trust,* strongly depends *on initial trust level* only, which again makes sense as that is what it refers to. Another interesting *statement type* is that of general motivational comments, where overall, the *initial trust level* seems to influence whether such a statement is used, whereas *PCL trend* does not have much impact. This is probably because most such general statements were positive in nature, so they were compliments, or they confirmed that it was going well. *Initial trust level* seems to influence whether these are more suitable than symptom progression.

#### Improving the Model

Looking at the empirical results, our model shows promise, but also that there is room for improvement. First, there was 1 situation in which the general messages outperformed the personalized messages. This means that for this situation, the personalized system needs to be improved. However, to do this properly, the first step would be to examine why the general motivation worked better. It is unclear whether this was truly, as suggested, because people prefer reassurance when trust is low even if symptoms are actually reducing. If this is the case, the likelihood table would need to be adapted for this situation.

**Figure 15:**
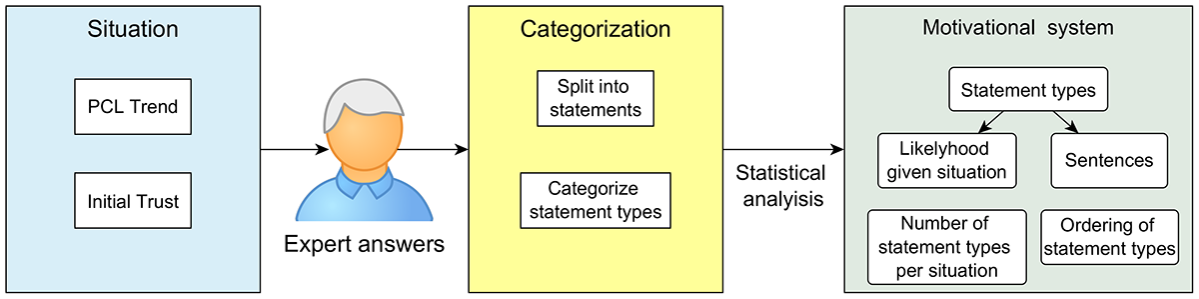
Different stages of building the motivational system. From the situation, we receive expert answers, which are then categorized, and then a statistical analysis follows, resulting in a system that matches statement types with situations using likelihood numbers and then chooses matching sentences. It also includes information on the number of statement types to include and the order in which to present them. PCL: post-traumatic stress disorder-check-list.

Besides this specific situation, general improvements could also be made. These improvements could be made at different stages of building the model. These stages can be seen in Figure 15. First, the model might be improved by providing more information about the situation. Adding variables such as stage in the therapy or personality might allow for a more fine-grained personalization. Another option would be to include more expert participants to create a larger corpus of sentences as well as making the likelihood table more stable. Our current model is based completely on the answers of these experts, so their role in building this model is large. Then, the categorization might be improved further as well. One option would be to let the experts rate the categorization or provide the statement types they believe they have used themselves. To improve the statistical analysis, 1 possible method would be to let the experts rate each other’s answers and use these as weights. Another method for improvement would be to analyze the order in which motivational statement types are used. Our current system orders the statements similarly for every situation, but perhaps, our input variables could be used to personalize this order as well. This would possibly improve the way the motivational messages are generated. All these options are aimed to eventually improve the content motivational system.

Something that is increasingly receiving attention in the literature is the goal to learn about the preferences of the user over time. However, this requires a measure of the success of the motivational message as well as enough time to learn. Given our current application domain, this might not be completely suitable. The only measure of success would be whether someone drops out of therapy, but that would mean no more messages are provided in any case. Moreover, participants only participate in 12 sessions, so by the time a learned model becomes good enough, the therapy might be over. Given the severity of PTSD and the importance of therapy, it is preferable to start with motivational messages, which are optimized beforehand.

### Limitations

To fully appreciate the system and results presented in this paper, it is important to also consider the limitations. Although the motivational message system is based on the patient’s situation, it only considers 2 parameters, namely, *symptom* trend and *initial trust*. However, other factors such as interpersonal differences in personality and gender could, for instance, also be used [32]. Moreover, the system currently does not take into account the patient’s stage in therapy, for instance, if they have just started or are near the end. However, this might also affect what statement types are most suitable to use in the motivational message [49]. Another limitation is that the data gathered from experts were all in Dutch, whereas the user study was performed in English. In both cases, this was the native language of our participants, and as Dutch and English are quite similar in structure, it was possible to make relatively direct translations. However, future work would be necessary to better study the effect of language on motivational effect. This ties in with possible cultural differences that might need to be reflected on in the content of the motivational messages.

In the user study design, all the participants were presented with all the different situations, something which would not happen in a real therapy. Responder bias might have caused participants to feel the need to reply differently to each situation, whereas some messages might actually have had the same effect. Although this might have caused more variance, that is, noise, it is unlikely to have caused any systematic bias as this was mitigated by randomization of the order in which participants were exposed to the situations. Another limitation is that the variables measured in this experiment relied on self-reports of the participant and responder bias might have, therefore, played a role. Moreover, they were asked to indicate what effect messages would have, given an imaginary situation. This imaginary aspect of our manipulation and measures could have affected our results. Similarly, the experiment was not performed with PTSD patients currently in therapy. However, we did not find indications that experience with exposure therapy influenced our results, which sheds a favorable light on the generalizability of the results. It is more difficult to say what the effect of the message system would be for a full therapy, as participants were only presented with situations in a single session. Further research would have to be done to study what results repeated motivation during the course of therapy would have. Such research could also shed light on the influence of a more interactive virtual agent. In the user study, video clips were used, but a more adaptive agent might further influence how motivation by this agent is perceived.

### Contributions

The main scientific contribution of the work presented in this paper is the motivational message system and its empirical evaluation. The motivational messages generated by this system could be used in other applications for PTSD therapy. Moreover, with minimal rewording of the messages, it might also be usable for exposure therapy systems for other disorders. Although we would not recommend using the exact statements and likelihoods for other domains, the underlying development approach could be applied to many other health care systems as well. An implemented version of the model described in this paper can be found in the study by Tielman et al [50]. Our study shows that a method involving the analysis of expert knowledge can be used to produce motivational messages that work to motivate users. The use of such an approach could be of benefit to other systems that currently rely exclusively on theory due to lack of data [36].

Similar systems could be applied to many different interventions, as shown by the broad range of mental health applications using motivational statements [8,22,51]. Moreover, our system sheds light on exactly how to motivate patients during therapy. The different statement types reveal what topics domain experts would address and that the patient’s personal situation has an effect on the type of language used. For instance, it was very common for experts to not just use purely motivational language but also to first explicitly refer to the user’s situation. Many experts used sentences to explain that negative feelings were normal, but would eventually go away, indicating that it is important to refer to a positive future. Moreover, the exact type of motivational statement did differ per situation, with, for instance, compliments being more suitable when people trusted in a good outcome, regardless of their symptoms, whereas phrases such as “go on like this” were used much more when symptoms were decreasing and trust was also good. These insights could be used to better tailor motivational messages in other applications as well.

### Conclusions

Google Assistant, Amazon Alexa, and Apple Siri are popular conversational agents that allow people to access a variety of services. Their capability and popularity seem to be growing. This paper shows how such agents could generate motivational messages personalized to the users’ situation and successfully increase their trust in a good outcome and motivation to continue. Moreover, it outperforms general motivational messages in several situations as well as making the user feel more heard by the system. This successful application shows how domain knowledge can be gathered from experts to build smarter technology. Especially in domains such as mental health care, where data on how to give therapy are not always available, such methods can be valuable. Moreover, given the importance of motivation in mental health applications in general, a suitable motivational message system could have the potential to increase adherence and reduce dropouts. Moreover, we hope, this will eventually even improve the overall effectiveness of e-mental health therapy.

## Abbreviations

ANOVA: analysis of variance
BCSS: behavior change support systems
eHealth: electronic health
e-mental: electronic-mental
MI: motivational interviewing
PCL: post-traumatic stress disorder-check-list
PTSD: post-traumatic stress disorder
TTM: transtheoretical model
